# Procalcitonin as new inflammatory mediator in cases of 1st episode drug naive major depressive disorder: a case-control study

**DOI:** 10.1192/j.eurpsy.2022.663

**Published:** 2022-09-01

**Authors:** S. El Sayed, S. Gomaa, Z. Gomaa

**Affiliations:** Faculty of Medicine-Mansoura University, Department Of Psychiatry,faculty Of Medicine, Mansoura University, Mansoura, Egypt

**Keywords:** Procalcitonin, inflammatory, Depressive

## Abstract

**Introduction:**

Procalcitonin (PCT) is the prohormone of calcitonin.Whereas calcitonin is only produced in the C cells of thethyroid gland as a result of hormonal stimulus, PCT is secreted by different cells from numerous organs in response to proinflammatory stimulation, particularly bacterial overactivity,also procalcitonin level might be elevated during the depressive episode as a result of inflammatory theory.PCT assessment is not fully studied in different psychaiatric disorders and particularly in major depressive disorder.

**Objectives:**

1-To study the level of Procalcitonin level in 1st episode drug naive major depressive disorder. 2-To investigate the relation between procalcitonin level and cognitive dysfunctions in these patients 3-To illustrate the role of PCT in psychopathology of Major depressive disorder

**Methods:**

1-Socio-demographic data of the target group of patients 2-Psychiatric evaluation using DSM 5 diagnostic criteria 3-Hamilton rating scale of Depression 4-Laboratory assessment of Procalcitonin level (PCT) using VIDAS® B·R·A·H·M·S PCT™ 5-Cogintive evaluation using novel battery of THINC-IT

**Results:**

1-Elevated level of Procalcitonin(PCT) in the targeted patients in comparison to control group 2-The level of PCT is positively associated with the cognitive dysfuctions reported in these patients. 3-The severity of depressive psychopathology is related positively to the elevated level of PCT

**Conclusions:**

Procalcitonin (PCT) assessment played an importnat role in the etiopathogenesis of 1st episode drug naive major depressive disorder,also it has a crucial role in the cognitive dysfunctions commonly reported in these patients

**Disclosure:**

No significant relationships.

